# Iodine Status and Consumption of Key Iodine Sources in the U.S. Population with Special Attention to Reproductive Age Women

**DOI:** 10.3390/nu10070874

**Published:** 2018-07-06

**Authors:** Kirsten A. Herrick, Cria G. Perrine, Yutaka Aoki, Kathleen L. Caldwell

**Affiliations:** 1 Division of Health and Nutrition Examination Surveys, National Center for Health Statistics, Centers for Disease Control and Prevention (CDC), Hyattsville, MD 20782, USA; yaoki@cdc.gov; 2 Division of Nutrition, Physical Activity, and Obesity, CDC, Atlanta, GA 30341, USA; cperrine@cdc.gov; 3 Division of Laboratory Sciences, CDC, Atlanta, GA 30341, USA; kcaldwell@cdc.gov

**Keywords:** iodine status, urinary iodine concentration, food group intake, dairy products, grains, soy, goitrogens, National Health and Nutrition Examination Survey

## Abstract

We estimated iodine status (median urinary iodine concentration (mUIC (µg/L))) for the US population (6 years and over; *n* = 4613) and women of reproductive age (WRA) (15–44 years; *n* = 901). We estimated mean intake of key iodine sources by race and Hispanic origin. We present the first national estimates of mUIC for non-Hispanic Asian persons and examine the intake of soy products, a potential source of goitrogens. One-third of National Health and Nutrition Examination Survey (NHANES) participants in 2011–2014 provided casual urine samples; UIC was measured in these samples. We assessed dietary intake with one 24-h recall and created food groups using the USDA’s food/beverage coding scheme. For WRA, mUIC was 110 µg/L. For both non-Hispanic white (106 µg/L) and non-Hispanic Asian (81 µg/L) WRA mUIC was significantly lower than mUIC among Hispanic WRA (133 µg/L). Non-Hispanic black WRA had a mUIC of 124 µg/L. Dairy consumption was significantly higher among non-Hispanic white (162 g) compared to non-Hispanic black WRA (113 g). Soy consumption was also higher among non-Hispanic Asian WRA (18 g compared to non-Hispanic black WRA (1 g). Differences in the consumption pattern of key sources of iodine and goitrogens may put subgroups of individuals at risk of mild iodine deficiency. Continued monitoring of iodine status and variations in consumption patterns is needed.

## 1. Introduction

Iodine is a trace element that is required for the synthesis of thyroid hormones, which are necessary for adequate growth, development, and metabolism [1]. Deficiencies can lead to significant health consequences across the age spectrum, including goiter (enlarged thyroid [2]), impaired mental functioning, and reduced productivity [3]. Serious adverse consequences can result from iodine deficiency during pregnancy, where the developing fetus experiences impaired neurodevelopment [4]. Cognitive disability can result, with the most severe outcome being cretinism [5]. Monitoring iodine status prior to and during pregnancy is therefore an important strategy for reducing cognitive disability. Since a developing fetus is solely dependent on maternal thyroid hormone early in pregnancy [6], and since 40% of pregnancies are unplanned [7], the iodine status of reproductive age women is a common proxy for women planning pregnancy.

The world’s oceans are the main reservoir of iodine, containing iodide ions, and organic and inorganic iodine species which are volatized from seawater to the atmosphere and then eventually deposited on land as components in rain water [8]. Iodine concentration in soil is known to be lower in inland areas than coastal areas, with additional variation due to soil’s capacity to retain iodine and other factors [8]. Iodine naturally enters the food supply through animals and plants grown on iodine replete soil and foods harvested from the oceans, i.e., fish, shellfish, and seaweed [5,9,10,11,12]. In the U.S., a significant proportion of iodine in the food supply comes from dairy products, which account for 60–70% of iodine intake from food sources among 6–12 year olds, and roughly 50% for adults [10,13]. The contribution of these food sources to total iodine intake may be slightly overstated, as they do not account for iodine from supplements, water, or iodized salt, which can contribute between 10–20% of iodine intake [14]. The iodine content in dairy milk can vary widely [15], depending on the iodine content in cattle feed, teat-dipping practices before and after milking [16], and the presence of goitrogens (substances that can block the uptake or utilization of iodine in cattle feed [17]). Other common food sources of iodine include grains and eggs [10,18].

Dietary supplements and iodized salt [19] are also sources of iodine. Among dietary supplements, prenatal vitamins are the most likely to contain iodine, as its inclusion in any dietary supplement is voluntary [20]. Table salt is voluntarily fortified with iodine in the U.S. at 45 mg iodide/kg [21], and no other food items are regularly fortified with iodine [22].

Roughly 90% of all iodine consumed is excreted in the urine [3] and thus, urinary iodine concentration (UIC) is a sensitive indicator for current intake [23]. UIC can be easily measured in a relatively inexpensive manner through the collection of a spot-urine sample [23]. The World Health Organization (WHO) defines the following median UIC (mUIC) concentrations of nutritional iodine sufficiency for a population: excessive iodine intake, ≥300 µg/L; more than adequate intake, 200–299 µg/L; adequate intake, 100–199 µg/L; mild iodine deficiency, 50–99 µg/L; moderate iodine deficiency, 20–49 µg/L; and severe iodine deficiency <20 µg/L [23]. During pregnancy, iodine requirements increase. The mUIC of 150–249 µg/L in pregnancy is associated with adequate iodine intake and a mUIC <150 µg/L is associated with inadequate intake [23].

The U.S has been considered iodine sufficient for decades [24,25]. However, population estimates of mUIC from the National Health and Nutrition Examination Survey (NHANES) have been decreasing, while remaining in the adequate range. Caldwell et al. reported a significant decrease in mUIC among the US population aged 6 years and over, from 164 µg/L (CI 154–173) in 2007–2008, to 144 µg/L (CI 132–154) in 2009–2010 (*p* = 0.001) [26]. Similar declines have been reported separately among men and women from 2001–2004 through 2009–2012 [27] and among pregnant women from the 1970s until 2007–2010 [28]. Iodine status may also be of concern among subgroups of the US population who report low consumption of dairy, eggs, and bread [18,29,30], key sources of iodine in the U.S. diet [10].

Beginning in 2011–2012, for the first time, NHANES has nationally representative data of urinary iodine status and food group consumption for non-Hispanic Asian persons. The goal of this analysis is to update estimates of iodine status for the US population and women of reproductive age (WRA). We present estimates of iodine status by subgroups of race and Hispanic origin, and examine dietary patterns associated with iodine status by race and Hispanic origin using the most recent data from NHANES 2011–2014.

## 2. Materials and Methods

### 2.1. Study Design

NHANES is a complex, stratified, multistage probability sample of the U.S. noninstitutionalized population, conducted by the National Center of Health Statistics (NCHS). Since 1999, NHANES has continuously collected and publicly released data on roughly 10,000 participants every 2 years. Participants receive a detailed in-home interview, followed by a dietary interview and a physical examination, which includes sample collection for laboratory tests, at a mobile exam center (MEC). Participants aged 18 and over provide consent, children and adolescents aged 7 to 17 years provide documented assent, and parental permission is obtained for those younger than 18 years. Non-Hispanic Asian, non-Hispanic black, and Hispanic individuals were oversampled for the 2011–2014 survey years [31]. The NHANES protocol was approved by the NCHS Research Ethics Review Board. The unweighted examination response rate for all participants in the 2011–2012 and 2013–2014 survey cycle was 69.5% and 68.5% [32].

### 2.2. Iodine Status

Spot urine samples were collected from a one-third sample of participants aged 6 years and over in the MEC. UIC was assessed using an Inductively Coupled Plasma Mass Spectrometer with Dynamic Reaction Cell Technology (ELAN^®^ DRC II) (PerkinElmer, Norwalk, CT, USA) [33,34,35]. Because iodine excretion is highly variable for an individual on a daily basis, UIC is a poor marker of individual iodine status, but individual variation is thought to even out within the population [36]. The WHO recommends using mUIC from spot urine samples to monitor the iodine nutritional status of a population [23]. Method quality parameters for this method have been described previously [24,26,33,34]. Briefly, absolute assay accuracy was verified by the analysis of National Institute of Standard Technology (NIST) 3668 Standard Reference Material.

### 2.3. Food Groups

Trained interviewers, using a computer-assisted dietary interview system that included a multiple-pass format with standardized probes [37], collected type and quantity of all foods and beverages consumed in the 24 h previous to the physical examination at the MEC (specifically from midnight to midnight). A second 24-h dietary interview was collected by telephone 3–10 days later. Since urinary iodine values directly reflect recent dietary iodine intake, the current analysis used data from the first 24-h recall only. Proxies, generally a parent, assisted children aged 6–11 years and study participants 12 years and over self-reported their dietary interview. Dietary interviews were assessed for reliability and completeness. Dietary interviews were considered unreliable if they are incomplete (i.e., all five steps in the AMPM are not finished) or if they included an eating occasion with missing information about the type and quantity of foods consumed [37,38]. We only used records deemed reliable in the current analysis (98%).

The USDA’s Food and Nutrient Database for Dietary Studies (FNDDS) [39,40] was used to code dietary intake data and calculate nutrient intakes from all of the foods and beverages participants reported consuming. The FNDDS does not capture the iodine content of foods and beverages because the iodine content in food can vary widely, dependent on location and farming practices. However, food and beverage sources that are known to be iodine-rich, such as dairy, grains, eggs, and fish, are captured and can be used to explore dietary patterns associated with UIC. Using the food-level files, we summed the amount consumed (g) of all food codes associated with dairy, grains, eggs, and fish, to calculate the total grams consumed per individual on a given day of each of these food types. We similarly calculated the total grams consumed of soymilk and soy products, per individual on a given day. Soy is a goitrogen—a substance that interferes with iodine uptake by the thyroid, and thus may render its consumers more susceptible to hypothyroidism from iodine deficiency [41]. Soy products included soymilk and soybean curd (tofu), soy sauce, soy nuts and edamame that were included in the “legumes, nuts and seeds” category of the food/beverage coding scheme. “Soy” identified in other categories typically represent a minor ingredient and we felt its inclusion with soy products would result in misclassification. For example, from the “Meat, poultry, fish, and mixtures” category, food code 27115000 has the description, “Beef with soy-based sauce”. This item was not included in the soymilk and soy products category created for the current analysis because the amount of soy consumed in the overall dish was likely a small fraction.

### 2.4. Supplements Contributing to Iodine Intake

Since 2007–2008, NHANES has included a 24-h dietary supplement recall, administered at the same time as the dietary recall. We used the day 1 24-h supplement recall to identify participants who had reported consuming a supplement that contained iodine in the previous 24-h. We dichotomized participants based on this reported consumption.

### 2.5. Use of Salt at the Table and in Food Prepartaion

Responses to the salt use questions administered during the 24-h recall allowed us to group participants by usage frequency for food preparation and at the table (never, rarely, occasionally, and very often). These questions do not ask specifically about iodized salt.

### 2.6. Covariates

Age is categorized into 8 groups for the US population (6–11, 12–19, 20–29, 30–39, 40–49, 50–59, 60–69 and 70+ years) and 4 groups for WRA (15–19, 20–29, 30–39, 40–44 years). We also used sex (male, female), race and Hispanic origin (non-Hispanic white, non-Hispanic black, non-Hispanic Asian, and Hispanic) to describe the data. Individuals with race and Hispanic origin classified as ‘other’ include those reporting multiple races, and are included in the overall estimates but not shown separately in the results. Pregnancy status for women aged 15–19 years, and 45 years and above is not included in the public use NHANES data files due to disclosure risk. We obtained this information through the Research Data Center (RDC, [42]).

### 2.7. Analysis

Statistical analyses were performed using SAS version 9.4 (SAS Institute Inc. Cary, NC, USA) and SUDAAN version 11.0 (RTI International, Cary, NC, USA). Survey design variables and sample weights, which account for differential probabilities of selection, nonresponse, noncoverage, and sample design, were used to obtain estimates representative of the noninstitutionalized US population. We used the day 1 dietary weights and adjusted for non-response in the urinary data (see below).

Guidance regarding selecting the most appropriate sample weight advise selecting “the weight of the smallest analysis subpopulation” [43]. The current analysis uses urinary iodine from a one-third sample of all eligible MEC participants, so it follows that the specific sample weights provided in the urinary iodine data file should be used when analyzing these data. However, the combination of urinary data with dietary data presents a unique situation where the general rule is not the best option. Sample weights are created to account for the complex survey design (including oversampling), survey non-response, and post-stratification [43]. Dietary weights are further adjusted to account for the additional non-response and to balance recalls across days of the week because of the differential allocation by weekdays (Monday through Thursday), Fridays, Saturdays and Sundays for the dietary intake data collection [44,45]. Thus, by using the weights found in the urinary iodine file, the additional adjustment made with the dietary weights are not accounted for. We used PROC WTADJUST in SUDAAN to adjust the dietary weights for non-response in the urinary data [46].

Using our adjusted weights, we calculated estimates for mUIC and Wald 95% confidence intervals. Neither SAS nor SUDAAN directly test the difference between medians. Using SUDAAN to account for complex survey design and weighting, we indirectly tested whether differences in mUIC by sociodemographic characteristics, salt, and supplement use were significant by first categorizing individuals as above or below the overall median. We then tested whether a below-overall-median proportion for any group differs from 0.5 with logistic regression. This indirect method is considered to be equivalent to testing whether medians varied by group [29]. We also tested the difference in mean consumption of dairy, grains, eggs, fish, and soy products. Differences (in means or proportions) were determined to be significant if *p*-values from adjusted Wald tests were less than 0.05. The hypothesis of no linear trend across ordinal variables was tested by using orthogonal contrast matrices (*p* < 0.05).

## 3. Results

In NHANES 2011–2014, 14,489 participants aged 6 years and over provided a complete and reliable dietary interview. Of these, 4744 participants were also in the 1/3 subsample who were eligible for urine iodine measurement. We excluded 68 participants because they did not have a value for urinary iodine, although they were eligible for urine collection (two common reasons for missing urinary measurements being urine collection refusal and insufficient urine volume), and 64 women who were pregnant or lactating, leaving a final analytical sample of 4613 participants, aged 6 years and over. For the analysis of WRA, the final analytic sample size was 901, after restricting to non-pregnant, non-lactating women, aged 15–44 years.

The mUIC in the US population was 133 µg/L (95% CI: 128, 141) (Table 1) in 2011–2014. By age, mUIC followed a quadratic trend (*p* < 0.05), with the lowest median among adults aged 40–49 years (107 µg/L, 95% CI: 97, 124), and highest medians observed among children aged 6–11 years (190 µg/L, 95% CI: 161, 211) and older adults aged 70 years and over (169 µg/L, 95% CI: 154, 197). Women had a significantly lower mUIC compared to men, 122 µg/L (95% CI: 112, 129) vs. 147 µg/L (95% CI: 136, 161) (*p* < 0.05). The only significant difference in mUIC by race and Hispanic origin in the US population was between non-Hispanic Asian individuals, (117 µg/L (95% CI: 104, 134)), and non-Hispanic blacks, (142 µg/L (95% CI: 127, 158) (*p* < 0.05)). The mUIC for non-Hispanic white and Hispanic individuals was 134 µg/L (95% CI: 125, 144) and 133 µg/L (95% CI: 123, 143), respectively. Among those who consumed a dietary supplement containing iodine, mUIC was significantly higher: 174 µg/L (95% CI: 152,193) compared with 127 µg/L (95% CI: 118, 133) among those with no consumption (*p* < 0.05). We found that mUIC did not vary by salt use at the table. However, mUIC was significantly higher among those reporting never using salt in food preparation: 172 µg/L (95% CI: 140, 201) compared to all other patterns of salt use: rare, 138 µg/L (95% CI: 127, 156), occasional, 128 µg/L (95% CI: 120, 137), and very often, 128 µg/L (95% CI: 117, 140) (*p* < 0.05).

The mUIC for US WRA, 15–44 years, in 2011–2014 was 110 µg/L (95% CI: 99, 124) (Table 2). We found that mUIC followed a significantly linear decline with age, from 128 µg/L (95% CI: 107, 170) in teenagers 15–19 years to 91 µg/L (95% CI: 75, 107) among women 40–44 years (*p* < 0.05). Among WRA, non-Hispanic Asian women had the lowest mUIC: 81 µg/L (95% CI: 54, 118), significantly lower than Hispanic (133 µg/L, 95% CI: 107, 163) women (*p* < 0.05). Differences between non-Hispanic Asian and non-Hispanic white women (106 µg/L, 95% CI: 92, 121) and non-Hispanic black women 124 µg/L (95% CI: 95, 159) were not significant. Differences in mUIC by supplement use were not significant. Among WRA with rare salt use at the table, mUIC was 122 µg/L (95% CI: 106, 144) significantly higher than those with occasional salt use (82 µg/L, 95% CI: 64, 119). WRA who reported rare salt use in food preparation has a significantly higher mUIC (117 µg/L, 95% CI: 106, 125) compared with occasional (100 µg/L, 95% CI: 85, 131) and very often use of salt in food preparation (107 µg/L, 95% CI: 97, 130) (*p* < 0.05).

Table 3 shows the mean consumption of key food and beverages sources of iodine in the US diet among individuals aged 6 years and over by race/Hispanic origin. Non-Hispanic white individuals had the highest consumption of dairy (249 g, (95% CI: 228, 271)) compared with non-Hispanic black (136 g, (95% CI: 119, 152)) and non-Hispanic Asian (180 g, (95% CI: 148, 213)) and Hispanic individuals (202 g, (95% CI: 183, 221)) (all pairwise comparisons, *p* < 0.05). Grain consumption was highest among non-Hispanic Asian persons (445 g, (95% CI: 417, 473)), followed by Hispanic (387 g, (95% CI: 360, 415)) and non-Hispanic black (331 g, (95% CI: 308,354)) and non-Hispanic white individuals (312 g, (95% CI: 296, 328)) (all pairwise comparisons, *p* < 0.05). There were no statistical differences in egg consumption by race and Hispanic origin; the average consumption was 23 g (95% CI: 21, 26). Both non-Hispanic blacks (23 g, 95% CI: 13, 34)) and non-Hispanic Asian individuals (21 g, (95% CI: 14, 28)) had higher fish consumption compared with non-Hispanic white (12 g, (95% CI: 7, 17)) and Hispanic individuals (10 g, (95% CI: 6, 14)) (all pairwise comparisons, *p* < 0.05). Consumption of soy products was highest among non-Hispanic Asian individuals (26 g, (95% CI: 18, 33)) compared to non-Hispanic white (7 g, (95% CI: 4, 11)), Hispanic (6 g, (95% CI: 2, 11)), and non-Hispanic black individuals (3 g, (95% CI: 1, 7)) (all, pairwise comparisons, *p* < 0.05).

Among WRA (Table 4), non-Hispanic black women consumed significantly less dairy (113 g, (95% CI: 83, 142)) compared to non-Hispanic white women (162 g, (95% CI: 136, 187)) (*p* < 0.05). Differences in dairy consumption between Hispanic WRA (154 g (95% CI: 123, 186) and other race and Hispanic ethnic groups and between non-Hispanic Asian women (119 g, 95% CI: 79, 159) were non-significant. The only significant difference in mean grain consumption was between non-Hispanic Asian women (388 g (95% CI: 314, 461)), compared to 293 g (95% CI: 253, 333) by non-Hispanic white women, (*p* < 0.05). Non-Hispanic black and Hispanic WRA consumed 308 g (95% CI: 269, 347) and 343 g (95% CI: 295, 391), respectively. There were no statistical differences in egg consumption by race and Hispanic origin; the average consumption was 22 g (95% CI: 17, 26) among WRA. Non-Hispanic black WRA consumed significantly more fish (27 g, (95% CI: 10, 44)) compared with non-Hispanic white (5 g, (95% CI: 2, 9) and Hispanic WRA (8 g, (95% CI: 3, 13)) (all pairwise comparisons, *p* < 0.05). Non-Hispanic Asian WRA consumed 24 g (95% CI: 5, 43) of fish. Non-Hispanic black WRA consumed significantly less soy (1 g (95% CI 0.1, 3)) compared with Non-Hispanic Asian 18 g (95% CI: 4, 32) and non-Hispanic white WRA (8 g (95% CI: 2, 14)) (all pairwise comparisons, *p* < 0.05). Hispanic WRA consumed 9 g (95% CI: 0.1, 16) of soy.

**Figure 1:**
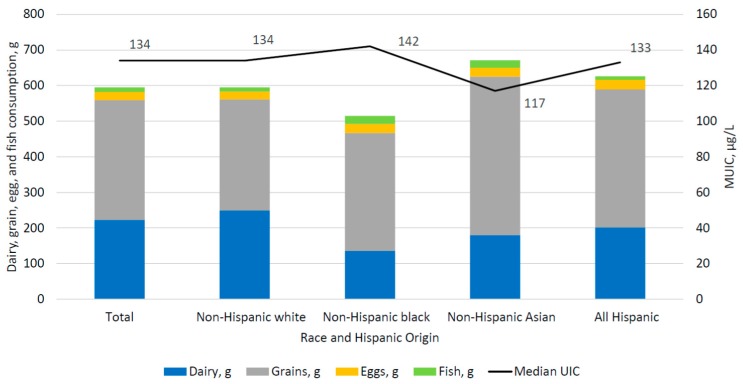
Median UIC and mean dairy, grain, egg, and fish consumption, by race and Hispanic origin among children and adults aged 6 and over, NHANES 2011–2014.

**Figure 2:**
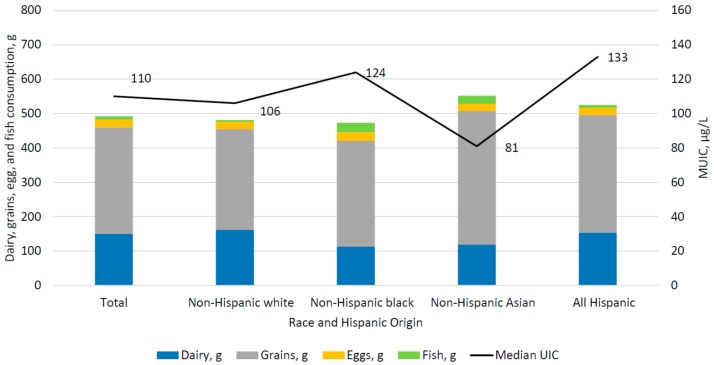
Median UIC and mean dairy, grains, egg, and fish consumption, by race and Hispanic origin among women aged 15–44 years, NHANES 2011–2014.

Figure 1 displays the juxtaposition of mUIC and the reported mean consumption of key food and beverage sources of iodine, by race and Hispanic origin among US children and adults aged 6 years and over. Figure 2 presents similar data for WRA. Differences in the consumption pattern of food sources of iodine are not reflected in differences in mUIC. Despite consumption of grains and dairy products in similar amounts to non-Hispanic whites, non-Hispanic Asian persons had lower mUIC compared to non-Hispanic whites.

## 4. Discussion

In this analysis of NHANES 2011–2014 we found that the general US population aged 6 years and above had a mUIC of 133 µg/L and WRA had a mUIC of 110 µg/L, suggesting iodine sufficiency, according to WHO criteria [23]. For the first time, we were able to present nationally representative data on the iodine status of non-Hispanic Asian persons. The low mUIC of non-Hispanic Asian WRA (81 µg/L), indicates mild iodine deficiency in this group according to WHO categorizations (50–99 µg/L) [23]. Previous analyses of NHANES have reported the mUIC of the general population as 164 µg/L in 2007–2008 and 144 µg/L in 2009–2010, and among WRA as 133 µg/L in 2007–2010 [26].

Despite the lack of a publicly available national nutrient database with iodine values for foods and beverages consumed in the US, there is consistent agreement that dairy, grains, eggs, and fish provide the bulk of iodine in the US diet [18,30,48], as assessed through mUIC. We found that non-Hispanic Asian WRA, consumed similar amounts of dairy and grains when compared to non-Hispanic black WRA; yet the mUIC for non-Hispanic Asian WRA (81 mg/L) was significantly lower than their non-Hispanic black (124 mg/L) counterparts, and indicative of mild iodine deficiency. This difference was more pronounced in the total population (aged 6 years and above), where non-Hispanic Asian individuals consumed greater amounts of dairy and grains, yet had a lower mUIC compared to non-Hispanic blacks, 117 mg/L vs. 142 mg/L, respectively. Asian individuals have been reported to consume more rice than other racial groups in the US [49]. Additionally, some reports have found bread, but not other grain products, to be a good source of dietary iodine. [11,18]. If the type of grain consumed is important, i.e., breads vs. rice, then the difference in mUIC between non-Hispanic Asian persons and non-Hispanic blacks, might be explained by different consumption patterns of rice vs. breads. Additional research may be needed to characterize the considerable variability in the iodine content of grain products; a limited and dated source comparing the iodine content of different grain products suggested that the iodine content in grains such as rice may not be as high as breads [50].

A novel finding of the current analysis is that non-Hispanic Asian individuals, who tended to have lower mUIC than other race/Hispanic origin groups, had higher consumption of soy products. Soymilk typically does not contain high amounts of iodine [51] compared to cow’s milk where incidental introduction occurs through cattle feed or iodophor disinfectants used in milking [11,15,16]. Soymilk and soy products contain substances that block uptake of iodine by the thyroid; thus, having lower mUIC and consuming more soy products may render these individuals doubly susceptible for hypothyroidism [41,52]. The relationship between iodine sufficiency and dietary intake in the US may be more nuanced than the frank presence or omission of certain foods, beverages, dietary supplements, and salt. Our results suggest that the pattern of consumption may be important to consider.

Salt iodization is considered the central strategy for eliminating iodine deficiency [53]. In the US, salt iodization is voluntary [54], and a recent analysis using Nielsen ScanTrack data identified only 53% of table salt sold in the US as iodized [55]. Additionally, the bulk of sodium intake in the US [~70%] comes from foods consumed outside of home [56,57], where the salt used in these foods is typically not fortified with iodine [25]. Puzzlingly, our results did not indicate a clear pattern between mUIC and salt use [at the table and in food preparation]. In fact, there was suggestion of higher mUIC with lower salt use. NHANES only captures salt use, but does not specifically capture whether the salt is iodized. Future efforts to capture this key source of iodine in the US diet may help to understand these findings.

The use of a dietary supplement containing iodine was positively associated with mUIC in the US population aged 6 years and above. A similar association between mUIC and use of a dietary supplement containing iodine was suggested for WRA; however, a significant difference was not detected, possibly because so few women in this age group consumed a dietary supplement with iodine. Like folic acid, iodine is critical for brain development in the early weeks of pregnancy when many women do not yet know they are pregnant [58]. Unlike folic acid, however, many multivitamin-multimineral supplements marketed to women planning to become pregnant do not contain iodine. There has been a modest increase in the proportion of supplements targeted to WRA that contain iodine; in 2009, roughly 51% of these supplements contained iodine [59], increasing to 61% in 2017 [20]. 

While our analysis did not include pregnant women, the Recommended Dietary Allowance for iodine increases from 150 µg/day when not pregnant to 220 µg/day during pregnancy. Thus, it is possible that without some changes in dietary intake, some women may not have sufficient iodine intake if they were to become pregnant. Severe iodine deficiency during pregnancy is detrimental for child survival and development, but the effects of mild-to-moderate iodine deficiency are less well defined. While challenging methodologically to assess this relationship because there is no established method for assessing an individual’s iodine status, mild iodine deficiency in pregnancy has been associated with poorer educational outcomes at 8–9 years among cohorts in Tasmania [60] and the United Kingdom [61] (mUIC during pregnancy 88 µg/L and 91 µg/L, respectively). Insufficient iodine intake during pregnancy, defined as dietary intake below the Estimated Average Requirement of 160 µg/d, has also been associated with language delays and behavior problems at 3 years of age [62].

This study is not without limitations. It is well documented that self-reported measures of dietary intake are subject to random and systematic error due to day-to-day differences in intake, systematic underreporting, difficulty remembering foods, and errors in the food and nutrient database that intakes are linked to [2,47]. As with any dietary study, there is the possibility of misclassification of individual foods into broad food groups. Additionally, iodine intake was not directly measured, but approximated by intake of food categories known to have variable iodine content. These limitations would tend to attenuate differences. Additionally, we used WRA as a proxy for women intending to become pregnant. As a group, women intending pregnancy may change behaviors to increase iodine intake through choosing supplements with iodine or using iodized salt at home; however, since 40% of pregnancies are unintended [7], this proxy seems reasonable. We limited the current analysis differences in iodine status by food consumption and did not investigate differences in supplementation use by race and Hispanic origin. Finally, the inverse association between iodine status and salt use in cooking or at the table could be related to social desirability, such that those who consumed a lot of salt chose not to report it.

The determination of iodine status as assessed by UIC also has challenges. The WHO definition of population iodine status does not include adjustment for creatinine [36]. However, iodine/creatinine ratios adjust for hydration levels, which can influence the concentration of metabolites, such as iodine, in urine. UIC also has a substantial day-to-day variation. Andersen el al. estimated that a minimum of 125 individuals is needed to reliably estimate the iodine status of a group [63]. In the current analysis, despite meeting data presentation standards for NCHS, some subgroups of WRA fell below this cut-off. Caution should be exercised when interpreting these values. Similar to dietary intake, urinary iodine experiences day-to-day variation. Like a single 24-h recall does not capture *individual* intake, neither does a single spot urine sample capture an individual’s iodine status, as measured by UIC [64]. We therefore avoided regression analysis that considers associations between dietary intake and UIC on an individual basis. Lastly, we were unable to test medians directly.

Despite merging two survey cycles, there was limited statistical power to identify differences in food group consumption by race and Hispanic origin among WRA. A separate issue is the considerable heterogeneity within race and Hispanic ethnic groups. For example, the non-Hispanic Asian group includes south Asian, Korean, Japanese, and Chinese, and eating patterns are likely to vary greatly among these subgroups, and the moderate iodine deficiency seen for among non-Hispanic Asian WRA may be more or less problematic in some racial subgroups.

Some analyses were beyond the scope of this study. Seaweed is a known source of iodine; however, the iodine content in seaweed is highly variable [25] and it is consumed infrequently in the U.S., making its estimation using NHANES data impractical. In addition to soy, other goitrogens exist, including chemicals that inhibit uptake of iodine by the thyroid (e.g., perchlorate, thiocyanate) [65]. Examination of these factors was beyond the scope of the current analysis, but given the suggestion of declining mUIC in the US population and lower mUIC levels among Asian persons documented here, future investigations considering exposure to these chemicals may be important.

The strengths of this analysis include the most recent, nationally representative estimates of iodine intake and mUIC for the US population, WRA, and for the first time, non-Hispanic Asian persons. We estimated mUIC by the important sources of iodine in the US diet: food and beverages, supplements, and salt use. Another novel contribution of this work is the exploration of soymilk and soy products as a source of goitrogens in the US diet that may block the utilization of iodine and contribute to mild iodine deficiency.

## 5. Conclusions

There is no overt sign of widespread iodine deficiency in the US, based on the observed mUIC from NHANES 2011–2014. However, non-Hispanic Asian WRA had a mUIC of 81 mg/L, indicating mild iodine deficiency in that subgroup. We found that mUIC did not follow the expected pattern of association with dietary intake of key food and beverage sources of iodine among all race and Hispanic origin groups. In the context of decreasing iodine status, variation in iodine status by race and Hispanic origin, and variation in dietary intake of key food sources of iodine, continued monitoring of iodine status and factors associated with iodine status is needed.

## Figures and Tables

**Table 1:** Median UIC (µg/L) of U.S. Children and Adults aged 6 years and over, by demographic and lifestyle characteristics, NHANES 2011–2014.

	*n*	mUIC (µg/L)	95% CI
Overall	4613	133	(128, 141)
Age ^1^, years			
6–11	698	190	(161, 211)
12–19	781	139	(126, 162)
20–29	569	120	(106, 138)
30–39	554	109	(99, 128)
40–49	522	107	(97, 124)
50–59	532	128	(111, 145)
60–69	504	138	(114, 164)
70+	453	169	(154, 197)
Sex			
Male	2344	147	(136, 161)
Female	2269	122 ^a^	(112, 129)
Race/Hispanic origin ^2^			
Non-Hispanic white	1671	134	(125, 144)
Non-Hispanic black	1112	142	(127, 158)
Non-Hispanic Asian	517	117 ^b^	(104, 134)
All Hispanic	1137	133	(123, 143)
Supplement containing iodine yesterday			
Yes	586	174	(152, 193)
No	4027	127 ^a^	(118, 133)
Salt used at the Table ^3^			
Never	1495	142	(130, 158)
Rare	1593	136	(128, 145)
Occasional	919	123	(106, 143)
Very Often	544	128	(119, 138)
Salt used in food preparation ^4^			
Never	291	172	(140, 210)
Rare	788	138 ^a^	(127, 156)
Occasional	1553	128 ^a^	(120, 137)
Very Often	1897	128 ^a^	(117, 140)

^1^ Significant quadratic trend in mUIC by age, *p* < 0.05; ^2^ Other race/Hispanic origin included in totals but not shown separately; ^3^ Respondents reporting “other salt” and “do not know” type of salt used were set to missing and excluded from the analysis of salt used at the table use, *n* = 61; ^4^ Respondents reporting “do not know” whether salt was used in preparation were set to missing and excluded from the analysis of salt used in preparation, *n* = 83; Notes: 95% Confidence Interval (95% CI), median Urinary iodine concentration (mUIC), µg/L. Different superscript letters represent a statistically significant pairwise difference (*p* < 0.05) between the estimate in the respective row the superscript appears in and ^a^ the first group, ^b^ the second group, listed under each covariate. Pregnant and lactating women aged 15–44 excluded (*n* = 64); Source: CDC/NCHS, NHANES 2011–2014.

**Table 2:** Median UIC (ug/L) of women 15–44 years, by demographic and lifestyle characteristics, NHANES 2011–2014.

	*n*	mUIC (µg/L)	95% CI
All	901	110	(99, 124)
Women of childbearing age ^1^, 15–44 years			
15–19	241	128	(107, 170)
20–29	268	119	(98, 137)
30–39	255	107	(88, 134)
40–44	137	91	(75, 107)
Race/Hispanic origin ^2^			
Non-Hispanic white	310	106	(92, 121)
Non-Hispanic black	186	124	(95, 159)
Non-Hispanic Asian	113 *	81	(54, 118)
All Hispanic	245	133 ^a,c^	(107, 163)
Supplement containing iodine yesterday			
Yes	54 *	147	(69, 297)
No	847	107	(98, 122)
Salt used at the Table ^3^			
Never	224	109	(85, 138)
Rare	341	122	(106, 144)
Occasional	215	82 ^b^	(64, 119)
Very Often	114 *	108	(87, 128)
Salt used in food preparation ^4^			
Never	39 *	133	(66, 208)
Rare	135	117	(106, 125)
Occasional	308	100 ^b^	(85, 131)
Very Often	415	107 ^b^	(97, 130)

^1^ Significant decreasing linear trend in mUIC with age, *p* < 0.05; ^2^ Other race/Hispanic origin included in totals but not shown separately; ^3^ Respondents reporting “other salt” and “do not know” type of salt used were set to missing and excluded from the analysis of salt used at the table use, *n* = 7; ^4^ Respondents reporting “do not know” whether salt was used in preparation were set to missing and excluded from the analysis of salt used in preparation, *n* = 4; * These estimates meet standards of data presentation for NCHS; however, one report suggests a minimum sample size of 125 individuals is needed for producing reliable estimates of UIC for groups [47] These estimates should therefore be interpreted with caution; Notes: 95% Confidence Interval (95% CI), median Urinary iodine concentration (mUIC), µg/L Different superscript letters represent a statistically significant pairwise difference (*p* < 0.05) between the estimate in the respective row the superscript appears in and ^a^ first group, ^b^ the second group, and ^c^ the third group, listed under each covariate. Pregnant and lactating women aged 15–44 excluded (*n* = 64); Source: CDC/NCHS, NHANES 2011–2014.

**Table 3:** Mean consumption of dairy, grains, eggs, fish, and soy products among children and adults aged 6 years and over, NHANES 2011–2014.

		Dairy (g)		Grains (g)		Eggs (g)		Fish (g)		Soy Products ^1^ (g)	
	*n*	Mean	95% CI	Mean	95% CI	Mean	95% CI	Mean	95% CI	Mean	95% CI
All	4613	223	(205, 240)	336	(324, 347)	23	(21, 26)	13	(9, 18)	8	(5, 10)
Race/Hispanic origin ^2^											
Non-Hispanic white	1671	249	(228, 271)	312	(296, 328)	22	(19, 26)	12	(7, 17)	7	(4, 11)
Non-Hispanic black	1112	136 ^a^	(119, 152)	331	(308, 354)	25	(21, 30)	23 ^a^	(13, 34)	3 ^a^	(1, 7)
Non-Hispanic Asian	517	180 ^a,b^	(148, 213)	445 ^a,b^	(417, 473)	25	(18, 32)	21 ^a^	(14, 28)	26 ^a,b^	(18, 33)
All Hispanic	1137	202 ^a,b^	(183, 221)	387 ^a,b,c^	(360, 415)	27	(23, 32)	10 ^b,c^	(6, 14)	6 ^a,c^	(2, 11)

^1^ Soy products included soymilk and soybean curd (tofu), soy sauce, soy nuts and edamame that were included in the “legumes, nuts and seeds” category of the USDA food/beverage coding scheme; ^2^ Other race/Hispanic origin included in totals but not shown separately, *n* = 176; Notes: 95% Confidence Interval (95% CI), Different superscript letters represent a statistically significant pairwise difference (*p* < 0.05) in mean consumption amount (g) between the respective row the superscript appears in and ^a^ non-Hispanic white, ^b^ non-Hispanic black, and ^c^ non-Hispanic Asian. Pregnant and lactating women aged 15–44 excluded (*n* = 64); Source: CDC/NCHS, NHANES 2011–2014.

**Table 4:** Mean consumption of dairy, grains, eggs, fish, and soy products among women aged 15–44 years, NHANES 2011–2014.

		Dairy (g)		Grains (g)		Eggs (g)		Fish (g)		Soy Products ^1^ (g)	
	*n*	Mean	95% CI	Mean	95% CI	Mean	95% CI	Mean	95% CI	Mean	95% CI
All	901	151	(133, 169)	310	(285, 336)	22	(17, 26)	10	(5, 14)	8	(4, 12)
Race/Hispanic origin ^2^											
Non-Hispanic white	310	162	(136, 187)	293	(253, 333)	21	(13, 29)	5	(2, 9)	8	(2, 14)
Non-Hispanic black	186	113 ^a^	(83, 142)	308	(269, 347)	25	(17, 34)	27 ^a^	(10, 44)	1 ^a^	(0, 3)
Non-Hispanic Asian	113	119	(79, 159)	388 ^a^	(314, 461)	21	(14, 28)	24	(5, 43)	18 ^b^	(4, 32)
All Hispanic	245	154	(123, 186)	343	(295, 391)	21	(12, 30)	8 ^b^	(3, 13)	9	(0.1,16)

^1^ Soy products included soymilk and soybean curd (tofu), soy sauce, soy nuts and edamame that were included in the “legumes, nuts and seeds” category of the USDA food/beverage coding scheme; ^2^ Other race/Hispanic origin included in totals but not shown separately, *n* = 47; Notes: 95% Confidence Interval (95% CI). Different superscript letters represent a significant pairwise difference (*p* < 0.05) in mean consumption amount (g) between the respective row the superscript appears in and ^a^ non-Hispanic white and ^b^ non-Hispanic black. Pregnant and lactating women aged 15–44 excluded (*n* = 64). Source: CDC/NCHS, NHANES 2011–2014.
